# Effects of Metabolism on Macrophage Polarization Under Different Disease Backgrounds

**DOI:** 10.3389/fimmu.2022.880286

**Published:** 2022-07-14

**Authors:** Jia-Xue Sun, Xiang-Hong Xu, Liping Jin

**Affiliations:** Shanghai Key Laboratory of Maternal-Fetal Medicine, Department of Biobank, Clinical and Translational Research Center, Shanghai First Maternity and Infant Hospital, School of Medicine, Tongji University, Shanghai, China

**Keywords:** macrophage, polarization, metabolism, maternal-fetal interface, pregnancy

## Abstract

Macrophages are versatile immune cells associated with various diseases, and their phenotypes and functions change on the basis of the surrounding environments. Reprogramming of metabolism is required for the proper polarization of macrophages. This review will focus on basic metabolic pathways, the effects of key enzymes and specific products, relationships between cellular metabolism and macrophage polarization in different diseases and the potential prospect of therapy targeted key metabolic enzymes. In particular, the types and characteristics of macrophages at the maternal-fetal interface and their effects on a successful conception will be discussed.

## Overview of Macrophages

### Origination of Macrophages

It is believed that macrophages belong to the mononuclear phagocytic system, mainly derived from bone marrow progenitor cells. When macrophages are within the bloodstream, they are called mononuclear cells. When inflammation or injury occurs, monocytes are recruited to tissues and induced into macrophages based on the condition of the local environment (1, 2). As a matter of fact, most adult tissue-resident macrophages are seeded during embryonic development, have self-renewal capacity and are maintained without depending on monocytes (2–4). The most majority of tissue-resident macrophages are derived from erythro-myeloid progenitors that develop in vitellus capsule embryos. However, the existence of tissue-specific macrophage progenitors that can promote the maintenance of adult macrophages has not been clarified (4). When the quantity of monocytes in the blood is reduced, those in the tissues are largely unaffected. However, monocytes and their progenitors can supplement classical tissue mononuclear phagocytes as needed (2). Embryonic macrophages are involved in tissue remodeling while monocyte-derived macrophages mainly play a role in host immune response (2). The activation status and the function of tissue macrophages under different stimuli are largely dependent on the local tissue microenvironment (3, 5). The importance of the local microenvironment has been identified through studies based on transcriptomics, epigenetics and open chromatin regions (3).

Currently, comparative studies of gene expression have been conducted in macrophages from the most commonly used cell sources: murine bone marrow, human peripheral monocytes, and human leukemic monocytic cell line THP-1, as well as those derived from induced pluripotent stem cells (iPSCs) which were generated from differentiated cells by upregulating pluripotency factors (Oct3/4, Sox2, c-Myc, and Klf4) (6). MCP1, IL6, TNF, IL10, CXCL12, IL1β and IL6 are expressed in human blood-derived macrophages; meanwhile, IL1β, TNFα, iNOS, IL12β, Arg1, VEGFA and IL10 are expressed in murine bone marrow-derived M1 and M2 macrophages (7). IL1 and CD36 are expressed in THP-1 cells *in vitro (* 7), which can be treated as a model of human macrophages when studing relatively simple biological processes, but cannot be used in more comprehensive immunopharmacology and drug screening programs (8). Human peripheral blood-derived macrophages and human induced pluripotent stem cell (iPSC)-derived macrophages showed similar gene expression patterns, reminding that iPSC-derived monocytes can be used as a credible cell source of human macrophages for *in vitro* studies (7, 9). Further studies are needed to determine how the heterogeneity of macrophages is reflected, whether the functions and activities of tissue-specific macrophages are different from those of peripheral migration-induced cells, and the concordance between *in vitro* and *in vivo* experiments.

### Classification of Macrophages

**Figure 1:**
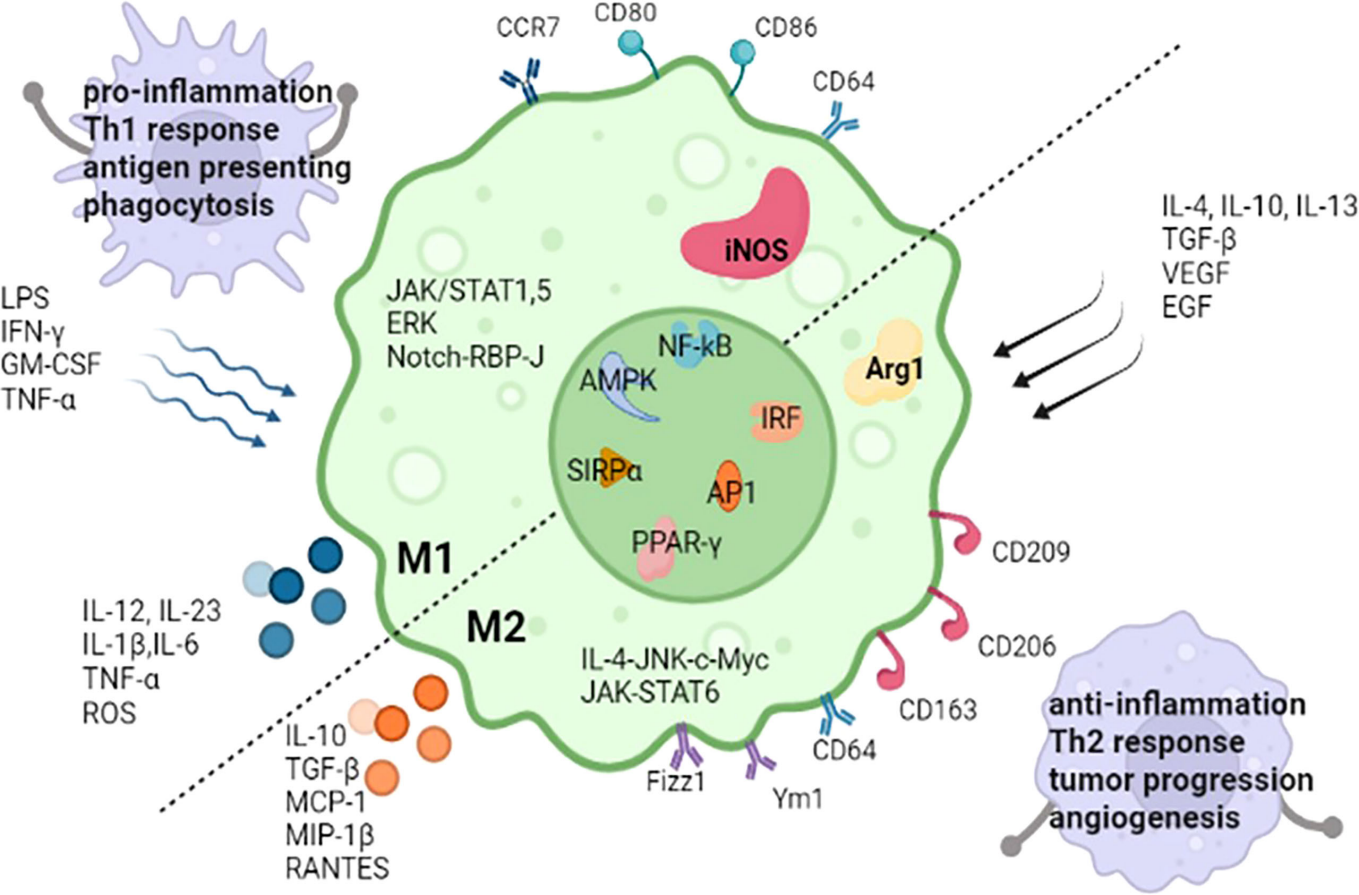
The common transcription factors and the differences of stimuli, markers, secreted cytokines, the main regulate pathways and functions between M1 and M2 macrophages. iNOS, inducible nitric oxide synthase; Arg1, Arginase 1; LPS, lipopolysaccharides; GM-CSF, granulocyte-macrophage colony stimulating factor; IFN-γ, interferon-gamma; IL, interleukin; TNF-α, tumor necrosis factor-α; TGF‐β, transforming growth factor‐β; ROS, reactive oxygen species; VEGF, vascular endothelial growth factor; EGF, epidermal growth factor; Ym1, chitinase 3-like 3; Fizz1, resistin-like-α; MIP-1β, macrophages inflammatory protein; MCP-1, monocyte chemo-attractant protein-1; RANTES, regulated on activation, normal T cell expressed and secreted; ERK, extracellular signal-regulated kinase; RBP-J, recombination signal-binding protein Jk; IRF, interferon‐regulatory factors; NF‐κB, nuclear factor‐κB; AP1, activator protein1; AMPK, adenosine monophosphate kinase; PPAR‐γ, peroxisome proliferator‐activated receptor‐γ; SIRPα, signal regulatory protein α. RBP-J, recombination signal-binding protein Jk.

Diversity and plasticity are hallmarks of macrophages. In response to IFN-γ/lipopolysaccharide(LPS) (10) or IL-4/IL-13, macrophages undergo M1 or M2 activation (11), which represent two extremes of a continuum of functional states. M1 macrophages have proinflammatory abilities and are able to initiate and maintain inflammatory reactions (12, 13), induce Th1 response activation (14), activate endothelial cells, amplify antigen presenting capacity (12, 14, 15), secrete proinflammatory cytokines and recruit other immune cells into inflammatory tissue. Nevertheless, M2 macrophages release anti-inflammatory mediators (16), support angiogenesis, induce adaptive Th2 immunity, refurnish and repair of scavenge debris and damaged tissue, take part in tumor progression, allergic reactions, and response to helminths (15). Some molecules are relative to both M1 and M2 macrophage polarization, such as NF‐κB (17), IRF, AP1, PPAR‐γ, AMPK and SIRPα (18). Some pathways are mainly involved in one polarization process of macrophages, JAK/STAT1 (12), JAK/STAT5 (19), extracellular signal-regulated kinase (ERK) (19), and Notch-RBP-J signaling pathway (18) take part in M1 polarization. JAK/STAT6 (12) and IL-4-JNK-c-Myc pathway (20) participate in M2 polarization. The common transcription factors and the differences in M1 and M2 macrophages are described in **Figure 1**.

Recent studies have found that the M2 macrophages are further sub-categorized into M2a, M2b, M2c, and M2d subtypes (21). M2a macrophages can be induced by IL-4 or IL-13, expressing high levels of arginase 1 (Arg1), FIZZ1 and Ym1 (22), playing a role in anti-inflammatory activity and tissue remodeling. M2b macrophages are activated by IL‐1 receptor ligand, immune complexes (ICs) and Toll-like receptor (TLR) agonists and produce both anti-inflammatory and proinflammatory cytokines, including IL-6, IL-10, CCL1, CD86, TNF-α and SpHK1 (23), playing a role in immunoregulation. The M2c subset of macrophages is induced by IL-10, TGF-β or glucocorticoids (24), secreting high levels of IL-10, TGF-β, CCL16, CCL18, MMP7, MMP8, and TIMP1, playing crucial roles in the phagocytosis of apoptotic cells (2) and wound healing (25). M2d macrophages, also called tumor-associated macrophages (TAMs), can be induced by IL-6 or combined exposure to adenosine 2A receptor (A2AR) agonists and TLR agonists. M2d macrophages secrete high levels of TGF-β, VEGF and IL-10, and low levels of IL-1β, IL-12 and TNF-α, taking part in tumor growth, angiogenesis and metastasis (26). It has been demonstrated that several stimuli in the tumor microenvironment, such as hypoxia, may contribute to the tumoral heterogeneity of TAMs. The integrated use of new technologies, such as single-cell RNA-seq, spatial transcriptomics, mass cytometry, and systems biology approaches, is promising to strongly reveal the tumoral heterogeneity of TAMs, potentially redefining TAMs with new valuable biomarkers (27). Excessive activity of either polarized phenotype is responsible for tissue damage, inflammatory disease, fibrosis, or tumor growth. Fortunately, macrophages sustain plasticity after activation and can change phenotypes according to the microenvironment (15).

Functional macrophage polarization has been reported *in vivo*, under physiological conditions (embryogenesis and pregnancy) and pathological conditions (chronic inflammation, infection and cancer). In some conditions, such as infection or pregnancy, macrophage polarizations express mixed or unique phenotypes. *in vitro* Because of the complexity of tissue macrophages, the *in vitro*-based M1/M2 dichotomous classification does not fully capture the classification of them. Some researchers have hypothesized that an M3 switch phenotype exists under the background of M1 and M2 phenotypes based on studies in lung diseases (28). M3 switch phenotype reprograms toward the anti-inflammatory M2 phenotype with proinflammatory stimuli, contrarily, M3 switches to proinflammatory M1 phenotype with anti-inflammatory stimuli. Therefore, understanding the role of coexisting phenotypes of macrophages and the mechanisms driving their dynamic modulation to adjust to the microenvironmental changes will be a key challenge in the coming years. The effort might provide a great prospective for designing macrophage-centered diagnostic and therapeutic strategies.

### Tissue Specific Macrophages: Maternal-Fetal Interface Macrophages

The maternal-fetal interface consist of various types of immune cells, such as decidual macrophages (dMϕs), natural killer (dNK) cells, T cells, dendritic cells, B cells and NKT cells. dMϕs account for a proportion of ~20% of decidual immune cells, and is the second most common immune cells during early human pregnancy (29). dMϕs are present in all stages of pregnancy and cannot be simply distinguished according to the M1 or M2 types. dMϕs constantly switch in the spectrum of continuous changes between the M1 and M2 types throughout the pregnancy. It has been found that dMϕs around the blastocyst are inclined toward M1 polarization before implantation. While trophoblast cells begin to invade the myometrium, dMϕ are inclined toward M1/M2 mixed-type polarization. M2 phenotype predominates during the late first trimester and maintenances of pregnancy during the second trimester. Then during the third trimester and parturition, M2 phenotype begins to decline and M1 phenotype again increases. Successful pregnancy requires that the dMϕs activation states remain regulated throughout pregnancy. Imbalanced M1/M2 dynamics are associated with complications, such as fetal growth restriction, preeclampsia and preterm delivery.

Different from dMϕs, which are primarily recruited from the maternal peripheral circulation, there is another homogeneous population of macrophages of fetal origin at the maternal-fetal interface, which are called Hofbauer cells (HBCs) (30). HBCs exist in the placental villi starting from Day 11 in mice and Day 18 in humans, persisting until parturition and representing a highly pleiomorphic cell population (31). HBCs phenotypically and functionally resemble M2 macrophages and are thought to have broad roles in immune regulation, placental morphogenesis, stromal water content and ion transport across the maternal–fetal barrier (32). *In vitro*Alterations of HBCs have been associated with several pregnancy disorders, such as Villitis and preterm delivery (33).

HBCs may be DC-SIGN+/CD163+, which may be connected with the relation between the high expression of IL-10 in the chorionic villi and the maintenance of immune regulation (34). CD28 expression has also been found in Hofbauer cells, which may adjust the immune function of leukocytes positively or negatively, and may have a very important influence on physiological or pathological processes (35). HBCs expressd higher levels of SPP1, PLIN2, HMOX1, CD36, and LYVE1, whereas dMϕs showed higher expression of HLA genes (HLA-DRA, HLA-DPA1, HLA-DRB1, HLA-DPB1, HLA-DQA1, and HLA-DMA) and invariant chain CD74, indicating strong antigen-presenting capacity of dMϕs. What’s more, dMϕ sspecifically expressed MS4A4A, STAB1, SEPP1, and MS4A7 (36). In a recent study, CD74 was regarded as a critical marker in Hofbauer cells. CD45^+^CD68^+^CD74^-^ cells were determined as HBC-like-1 cells and CD45^+^CD68^+^CD74^+^ cells were determined as HBC-like-2 cells (37). The expression of these markers is regulated by environmental signals, such as cytokines and hormones,. Meanwhile, it is suggested that differential epigenetic regulatory patterns might be critical for the functional and characteristic differences between dMϕ and HBCs (38). Epigenetic patterns will give clues for further understanding of the immunological characteristics of dMϕ and HBCs during human pregnancy.

## Metabolic Signature of Macrophage Polarization in Physiological and Pathological Situations

**Figure 2:**
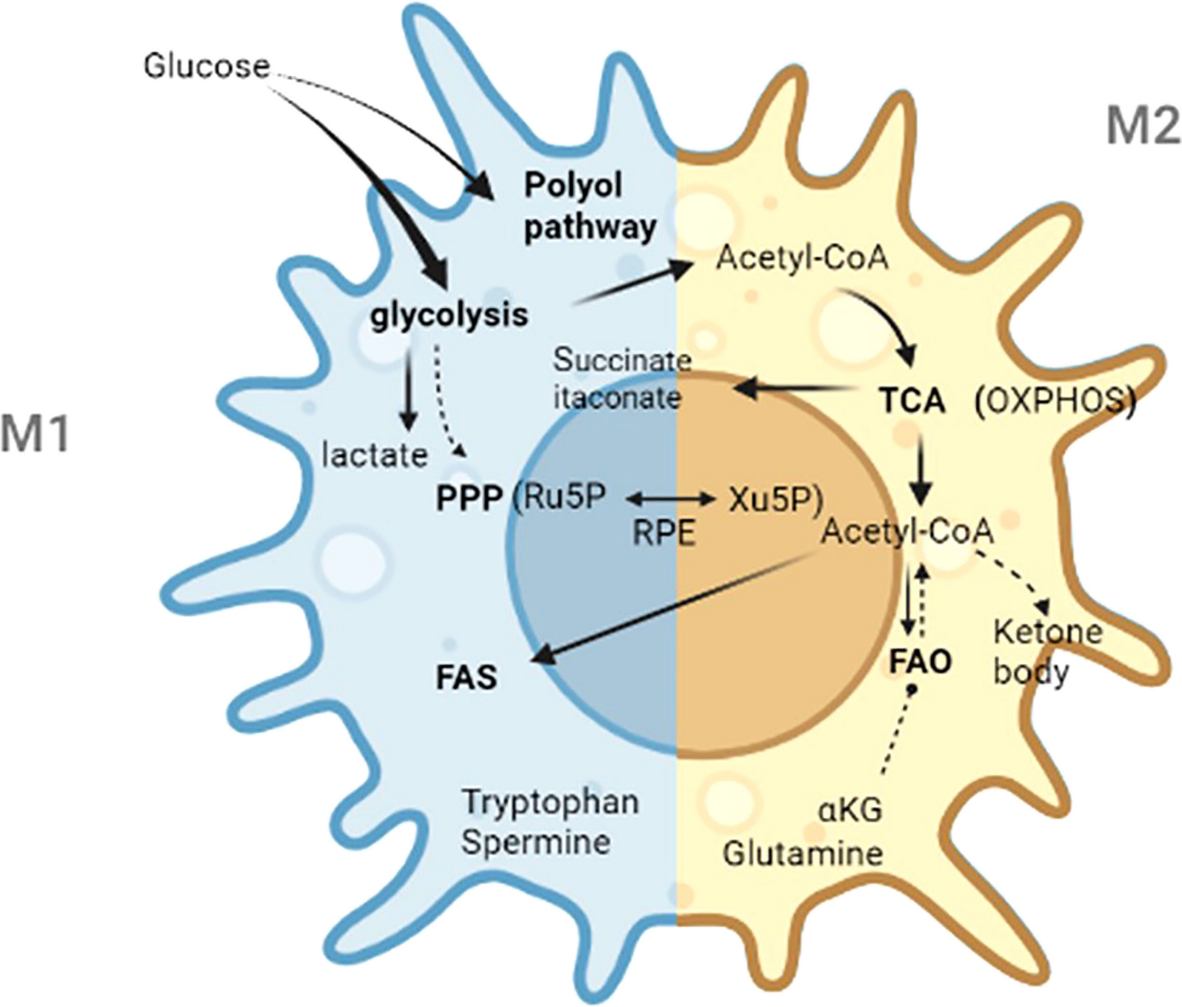
The main metabolic pathways and crucial metabolites in macrophages. Ru5P, d-ribulose-5-phosphate; Xu5P, d-xylulose-5-phosphate; RPE, ribulose 5-phosphate 3-epimerase; PPP, pentose phosphate pathway; TCA, tricarboxylic acid; FAS, fatty acid synthesis; FAO, fatty acid oxidation; Acetyl-CoA, acetyl-Coenzyme A; OXPHOS, oxidative phosphorylation; α-KG, α-ketoglutarate.

In the 1920s, Warburg reported the “Warburg effect” in which cancer cells “ferment” glucose to produce lactic acid in aerobic environment, whereas noncancer cells rely primarily on oxidative phosphorylation in the presence of adequate oxygen levels (39). Since then, studies have confirmed lipopolysaccharide which is the cell wall components of gram-negative bacteria can stimulate aerobic glycolysis and produce the Warburg effect in macrophages (40). In 2011, Mathis and Shoelson introduced the term immunometabolism, which was defined as the interaction between inflammation and metabolic diseases (40). Recently, the term immunometabolism has been given new meanings that incorporate the following components: contributions of metabolic pathways to the development, maturity, destiny, and behavior of immune cells; the changes in intracellular metabolic pathways that alter the functions of immune cells; and the metabolic reprogramming of immune cells (39). Immunometabolism opens new perspectives for modulating immune responses. Thus, targeting metabolic machineries is a potential treatment for immune-related diseases (17). This review mainly describes the metabolic characteristics of activated macrophages, the roles of specific metabolites, and the crucial steps in metabolic processes. In addition, metabolic features in decidual macrophages were summarized. The main metabolic specialties of M1 and M2 macrophages are exhibited in **Table 1**. The main metabolic pathways and crucial metabolites in macrophages are shown in **Figure 2**.

**Table 1:** The main metabolic characteristics in M1 and M2 macrophages.

Metabolic pathways	M1 macrophages	M2 macrophages
Glycolysis	Elevated glycolysis (41–43)	1) HIF-1α-dependent glycolysis (44) 2) Inhibited glycolysis (45)
PPP	1) Increased PPP (46) 2) Increased expression of NADPH-dependent ROS (47)	Restricted PPP (48)
TCA cycle	1) Two broken points (49, 50) 2) Accumulation of citrate and succinate (4, 51) 3) Production of itaconate (52, 53) 4) Generation of ROS (54, 55)	Entire TCA cycle provides ATP (56)
Lipid Metabolism	Increased FAS (57, 58)	FAO is essential (59)
Amino Acid Metabolism	1) Upregulated iNOS (60) 2) Reduced α-KG/succinate (61)	1) Upregulated arginase expression (60) 2) Rised α-KG/succinate (61)

HIF-1α, hypoxia inducible factor-1α; PPP, pentose phosphate pathway; ROS, reactive oxygen species; NADPH, reduced form of nicotinamide-adenine dinucleotide phosphate; FAS, fatty acid synthesis; TCA cycle, tricarboxylic acid cycle; FAO, fatty acid oxidation; ATP, adenosine-triphosphate; iNOS, inducible nitric oxide synthase; α-KG, α-ketoglutarate.

### Glycometabolism in Macrophages

#### Glycolysis

Glycolysis includes aerobic and anaerobic glycolysis. Pyruvate is the end product of glycolysis (62). Glycolysis not only provides rapid production of ATP, but also produces metabolic intermediates, including material used for the biosynthesis of nucleotides and proteins in macrophages (63). When LPS-inducible glycolysis is attenuated in macrophages, macrophages switch to the M2 phenotype and is accompanied by a decrease in the inflammatory response (41). However, elevated glucose levels could enhance the expression of inflammatory factors, such as IL-6, IL-1β and TNF-α, in monocytes (42). TAMs are a heterogeneous cell population dominated by M2-type macrophages, corresponding to the increased mitochondrial respiration and reduced glycolysis (64). It has been shown that chloroquine, a proven antimalarial drug, reprogrammed the metabolism of TAMs from oxidative phosphorylation to glycolysis and switched TAMs to the tumor-killing M1 phenotype (65). What’s more, glycolysis contributes to the proinflammation of macrophages in adipose tissue of obesities (66). M1 macrophages use glycolysis to rapidly kill other cells (43), while M2 macrophages obtain energy from mitochondrial oxidative phosphorylation (OXPHOS) and fatty acid oxidation (FAO) (4, 49, 67). Glycolysis is pivotal to M1-type macrophages, but its effects on M2-type macrophages remain unclear. While glycolytic activity is effectively suppressed, there is no effective suppression of the expression of M2 differentiation markers, intracellular ATP levels, oxidative phosphorylation and STAT6 phosphorylation (68). A recent study found that HIF1α-dependent glycolysis is associated with M2 macrophage differentiation, indicating that glycolysis is also essential to the M2 macrophages polarization (44). Mitochondrial elimination, also known as mitochondrial autophagy is the process of eliminating damaged mitochondria (69). It has been found that mitochondrial elimination is essential for the glycolytic switch during macrophage polarization toward the M1 phenotype (70).

There are three irreversible reactions catalyzed by hexokinase, 6-phosphofructokinase 1 (PFK1), and pyruvate kinase (PK) in the glycolytic pathway. These enzymes are three regulatory points of the glycolytic pathway. Recent studies have mainly focused on the isozymes of these enzymes. For example, researchers found that glucose uptake and the expression of proinflammatory cytokines were attenuated by inhibiting hexokinase 2 (HK2) expression in macrophages (71). Likewise, targeting inhibition the expression of glycolysis limiting enzyme PFK-m by miR-21 was associated with the suppression of macrophage glycolysis (72) Pyruvate kinase M (PKM) is glycolytic enzyme, containing two isoforms (PKM1 and PKM2), and also take part in the metabolic changes in activated macrophages. PKM1 converts phosphoenolpyruvate to pyruvate, which is the final step of glycolysis. Pkm2 was identified as a partner of proinflammatory Hdac7 and Pkm2-Hdac7 complex acts as an immunometabolism signaling hub in macrophages. Disrupting the Pkm2-Hdac7 complex attenuates LPS-inducible glycolysis and the accompanying inflammatory responses (41). From above findings, we can see that the regulation of the isozymes is crucial to the process of glucose metabolism and is a promising field for future research.

Lactate has been considered a waste product of glycolysis for decades. Studies in the recent years have shown otherwise. Lactate is an active signaling metabolite working through transporter- and receptor-mediated signaling, which directly regulate functional polarization (73). It has been demonstrated that cancer cell-deprived lactate is a Gpr132 activator which stimulates the macrophage M2 phenotype in a Gpr132-dependent manner (74). In addition, tumor-derived lactate is sufficient to increse the expression of M2-labeled MGL1, FizZ1 and MGL2 in BMDMs (75). The results in the study conducted by Ohashi et al. showed that more macrophages aggregate at the tumor site when the intratumoral concentration of lactate is lower; however, a higher concentration of lactate inhibits monocyte migration *in vitro (* 76). It is possible to say that the effects of lactic acid on immunity depend on the concentration of lactate. Hyperchloremia and lactic acidosis are two common metabolic acidosis, both of which are relative to distinct patterns of immune response. HCl is intrinsically proinflammatory through increased LPS-induced NF-κB DNA binding and NO release. Conversly, lactic acid significantly decreases LPS-induced IL-6, NO and IL-10 expression in a dose-dependent manner and inhibits LPS-induced NF-κB DNA binding (77). It is suggested that lactic acidos has an anti-inflammatory effect. From the functional comparison of these two situations, lactic acid possibly acts as a regulator unrelated to acidity. In addition, blocking the lactic acid signaling pathway can directly improve macrophage functions, even if the concentration of lactic acid in the tumor is not reduced (78). The pathway regulating lactic acid production can also influence the polarization of macrophages. Lactic acid clearance can be used for synergistic immunotherapy of tumors (79). Overall, we can predict that lactic acid is associated with M2-like macrophage polarization as a signaling mediator or through the regulatory pathway of its production. Lactate and the related signaling pathways reveal potential new therapeutic targets for disease amelioration.

#### Pentose Phosphate Pathway

PPP branches from glycolysis and contributes to inflammatory responses in macrophages (49, 57). The PPP contributes to amplifying the specific effector functions of LPS-activated macrophages, mainly providing two types of materials: pentose phosphates, which can participate in nucleotide and amino acid metabolism, and nicotinamide-adenine dinucleotide phosphate (NADPH), which is a critical factor for NADPH oxidase-dependent ROS (47). Nucleotide metabolism can be involved in the production of active substances, which are closely related to the function of macrophages. ROS plays a crucial role in the activation and maintenance of M1 macrophages, because ROS has strong oxidative characteristics and can damage intracellular constituents, such as nucleic acids, proteins and lipids. Mitochondrial ROS-induced lysosomal dysfunction contributes to M1 macrophage polarization (80) under diabetic conditions (81) and abamectin-induced cytotoxic disposition (82). Additionally, bacterial killing in macrophages is damaged when mitochondrial ROS (mROS) levels are attenuated (83). Researchers found that intracellular ROS production was significantly attenuated when the PPP was restricted, while M2 phenotype macrophage polarization was sensitized (48). Surprisingly, ROS also takes part in the anti-inflammatory reactions of macrophage phenotypes (84). In addition, glucose 6-phosphate dehydrogenase (G6PD) is highly active in macrophages and is the key enzyme in the PPP. The generation of ROS can be promoted by G6PD in macrophages under stressful conditions (80). In another trial, researchers found that proinflammatory responses were suppressed after the inhibition of G6PD in macrophages (85). It is reported that overexpression of G6PD enhanced the activation of the p38-MAPK and NF-κB signaling pathways and promoted the production of proinflammatory cytokines and ROS in a macrophage cell line (86, 87). Thus, the modulation of G6PD activity appears to provide a novel method for researchers to regulate macrophage activity and develop alternative therapies.

#### Tricarboxylic Acid Cycle

The TCA cycle, also named the citric acid or the Krebs cycle, uses a series of chemical reactions to generate energy in aerobic organisms (88). M2 macrophages can obtain enough ATP from OXPHOS through the TCA cycle. By contrast, M1 depends on ROS and intermediate metabolites (56). Mitochondrial ROS promotes macrophages swift to an inflammatory type through damaging the autophagy-lysosome system (81). OXPHOS deficiency in mitochondria is a feature of M1 macrophages. There are two breakpoints in M1 macrophages (49, 50): one breakpoint causes the accumulation of citric acid at the level of isocitrate dehydrogenase (IDH), which produces itaconate, and the other breakpoint causes the accumulation of succinate at the level of succinate dehydrogenase (SDH), which in turn induces HIF-1α mediated upregulation of glycolysis and IL-1β production (4, 51). In addition, SDH drives ROS generation (54, 55).

Citrate is reported to support both proinflammatory and anti‐inflammatory macrophage functions, which are mainly shown by citrate’s mediated products. The accumulation of citric acid increases NADPH through ATP‐citrate lyase (ACLY)-mediated reactions, which directly act on NADPH‐dependent inducible nitric oxide synthase (iNOS) and increase the production of NO (89). NO was shown to be able to induce M1 marker expression on peritoneal macrophages under Con-A treatment (90). Remarkably, NO has the ability to inhibit the mitochondrial electron transport chain (ETC). Researchers have demonstrated that OXPHOS during M1 polarization can be repressed by NOS-derived NO in bone-marrow-derived macrophages (BMDMs) (91). What’s more, inhibiting NO production in mitochondrial contribute to macrophage M2 type convertion (67). In addition, NO modulates the productions of citrate and succinate, which are TCA cycle metabolites and inflammatory mediator, respectively (51). There is a complex relationship between metabolites. In further explorations, the network of metabolites should be taken into account.

Itaconate is also a vital metabolite rooted in redundant citrate and is synthesized through the intermediate cis-aconitate produced by cis-aconitate decarboxylase under regulation of immune responsive gene 1 (IRG1) (52, 53). In addition, some researchers found that IDH activity and itaconate synthesis were inhibited by endogenous type I IFN-driven IL-10 *via* in LPS-macrophages (92). At present, itaconate and itaconate derivatives, 4-octyl itaconate (4OI), dimethyl itaconate (DI), and 4-monoethyl itaconate (4EI), are mainly studied. Researchers found (93) that OI alleviated LPS-induced acute lung injury. Activation of nuclear factor erythroid 2-related factor 2 (NRF2) may contribute to the anti-inflammatory and antioxidant effects of OI. However, Sun, K. A., et al. (94) found that endogenous itaconate does not take part in inflammation induced by particulate matter (PM) or activation of NRF2 in macrophages either *in vitro* or *in vivo*. Conversely, OI attenuated PM-induced inflammation in macrophages. In another trial, DI could suppress the inflammatory responses of macrophages by triggering NRF2 (95). Meanwhile, DI increased the production of NRF2 and its downstream factors NQO-1 and HO-1 in sepsis (96). In addition, researchers found that DI is not metabolized into itaconate intracellularly (97). Recently, some researchers (98) confirmed that nonderivatized itaconate can efficiently concentrate in macrophages, and can be used for mechanistic studies through comparing unmodified itaconate and commonly used itaconate derivatives. By contrast, many of the ester derivatives were not detected with similar results as expected for nonderivatized itaconate. Beyond this, the results represented that itaconate should be regarded as an immunoregulatory metabolite rather than a simple immunosuppressive metabolite. Therefore, more research is required to identify the features and moderating effect of itaconate and its derivatives.

Succinate is synthesized from α-ketoglutarate (51). Extracellular succinate is sensed by succinate receptor 1 (SUNCR1) and works as a signaling metabolite. The activation of SUCNR1 in macrophages take part in anti-inflammatory responses (99) and can polarize macrophages into TAMs in the tumor microenvironment (100). Moreover, succinate stimulates osteoclastogenesis in osteoclastic lineage cells *in vitro* and *in vivo* (101). In addition, succinate stabilizes hypoxia-inducible factor-1α (HIF-1α) (102), a transcription factor, which plays a crucial role in mediating aerobic glycolysis and M1 differentiation (103). Moreover, researchers have found that TAMs enhance aerobic glycolysis by stabilizing HIF-1α protein. In diabetic nephropathy, TAB1/TAK1 can activate NF-κB in bone marrow mesenchymal stem cells to upregulate HIF-1α activity and enhance glycolytic metabolism (104). In another study, intraperitoneal exudate macrophages of HIF1α -/- mice showed significantly reduced glycolytic activity compared to those of wild-type mice; this reduced glycolytic activity was associated with a reduced proton production rate (PPR, a value used to indicate the cellular respiration rate) and glucose transporter 1 (GLUT1) expression. In addition, these peritoneal exudate macrophages (PEMs) from HIF1α -/- mice showed lower secretion of proinflammatory cytokines and iNOS expression than those from wild-type mice (44). In glucose metabolism of macrophages, GLUT1 is a key regulator. The expression of proinflammatory cytokines (IL-6, IL-1B and TNF-α) and glucose uptake are obviously increased when GLUT1 is overexpressed in macrophages, even in the absence of stimulus-specific activation (105). It has also been reported that HIF-1α affects glycolysis in macrophages in an AMPK-dependent manner (106). In addition, HIFs are key effector molecules regulating the metabolism and functional reprogramming through the PDPK1/Akt/mTOR pathway under neuroinflammatory conditions in M1/M2 microglia (107). HIF-1α is closely related to energetic metabolism and improves the activity of glycolytic enzymes such as 6-phosphate fructose-2-kinase/fructose-2, hexokinase-1 (HK1), lactate dehydrogenase A (LDHA) and 6-bisphosphatase 3 (PFKFB3) (108). Overall, HIF-1α participates in the metabolism of macrophages and regulates the switching of phenotypes; therefore HIF-1α is a potential intervening target to macrophages.

### Lipid Metabolism in Macrophages

Fatty acid synthesis (FAS) takes part in the inflammatory reaction and signaling in macrophages (57). The proinflammatory response in macrophages can be inhibited through restraining fatty acid synthase (FASN), which is a key enzyme of FAS that catalyzes the production of long-chain fatty acids (58). Lipid droplets (LDs) is a feature of foamy macrophages that have been found in many diseases. In addition, polyunsaturated fatty acid (PUFA) metabolism in macrophages also influences pathological processes. In cardiovascular diseases, purified n-3 FA supplementation may be a potential strategy fortreatment and prevention (109). Therefore, understanding how immune cells handle and synthesize PUFAs is important. During macrophages activating, SREBPs and LXRs, the two master transcription factors of FAS, are upregulated. Upregulation in SREBPs enhances the maturation of pro-inflammatory precursors and causes macrophages to differentiate toward the pro-inflammatory M1 phenotype. In contrast, LXRα promotes cells towards an M2 phenotype (110).

FAO is essential in functional M2 macrophages by enhancing the secretion of IL-1β (59). Treatment with LPS attenuates the expression of inflammatory genes in macrophages when FAO is suppressed (111). FAO plays a crucial role in activation of NLRP3 inflammasome of M1 macrophages (112). It is implied that enhancing FAO in macrophages may be an underlying therapeutic method for patients with obesity, type 2 diabetes (113) or cancers (114). However, it remains unclear whether the increase in FAO correlates with M2 polarization (115). IL-4-induced polarization is not affected by the inhibition of FAO (116). The two subtypes of carnitine palmitoyltransferase (CPT) system, CPT1 and CPT2, are essential to the β-oxidation of long-chain aliphatic acid in mitochondria. Lacking CPT2, macrophages are unable to finish β-oxidation of fatty acids, but they can be polarized to M2 phenotype after stimulated by IL-4 *in vitro* and *in vivo* (117). Moreover, the CPT-1 inhibitor etomoxir in low concentrationcould suppress CPT-1 without changing M2 polarization.i However, high concentration of etomoxir can inhibit M2 polarization without affecting the activity of CPT-1 (56). Macrophage FAO likely plays a correlative rather than causative role in systemic metabolic dysfunction (118). Further exploration is needed to determine which is the dominant factor, the switchof cell phenotypes or the changes of metabolic transformation.

### Amino Acid Metabolism in Macrophages

**Figure 3:**
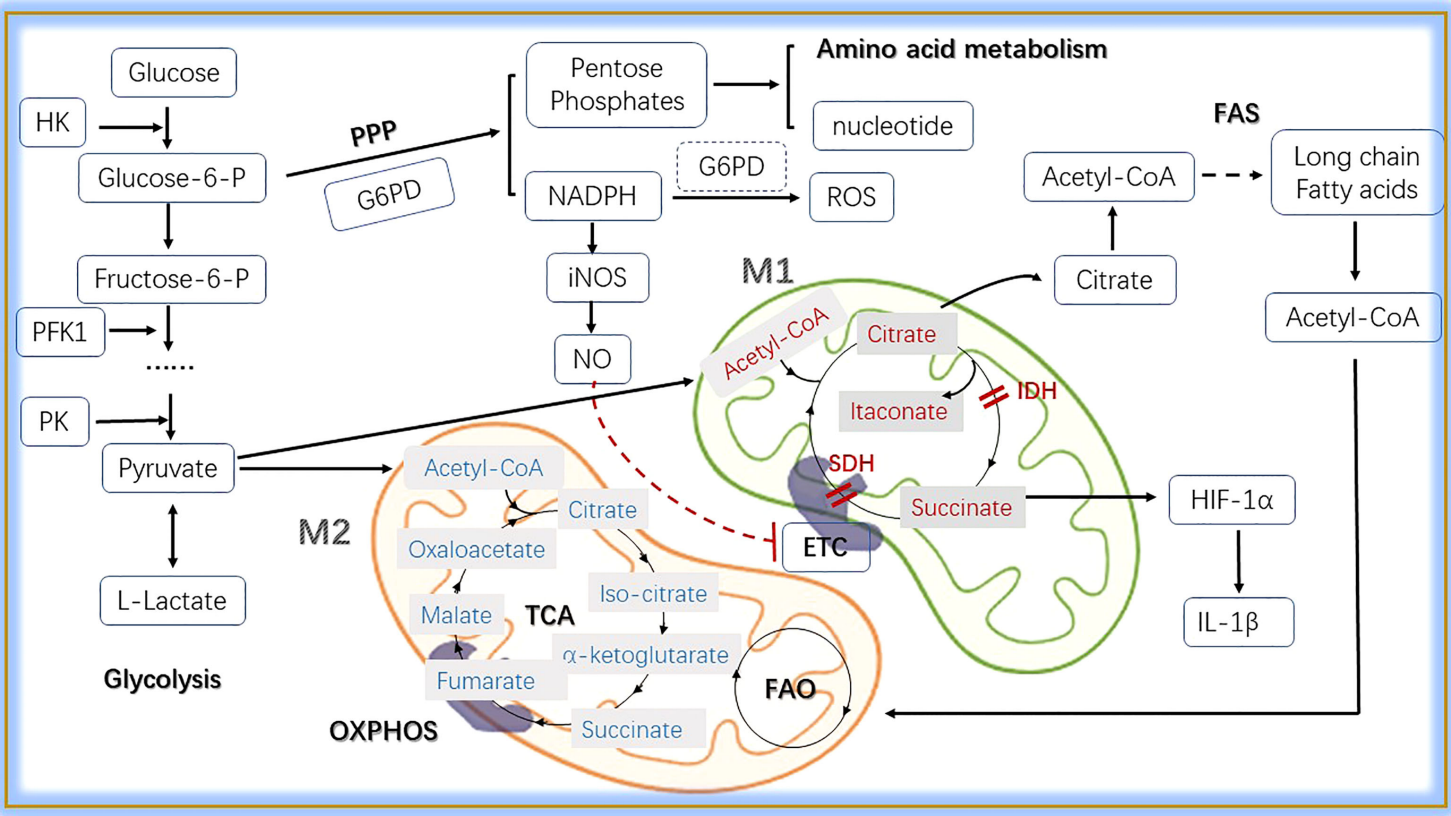
The main metabolic pathways in macrophages. In the cytosol, glucose is converted into L-lactate by glycolysis, in which HK, PFK1 and PK are key enzymes. In the progress of glycolysis, glucose-6-P can be shunted to PPP pathway sustaining pentose phosphates and NADPH production. Pentose phosphates are used for the synthesis of amnio acid and nucleotide, while NADPH contributes to the production of ROS and NO. Pyruvate is induced to lactate in hypoxic conditions, whereas decarboxylated into acetyl-CoA within the mitochondria in normoxic conditions. Here, acetyl-CoA enters into the TCA cycle, providing reducing agents to the ETC through OXPHOS to generate energy. Citrate, a metabolite in TCA, participates in fatty acid synthesis when exported to the cytoplasm. Acetyl-CoA produced from FAS can be transferred into mitochondria and take part in the FAO. In M1 macrophages, two breakpoints cause the production of itaconate and the accumulation of succinate that induces HIF-1α mediated upregulation of glycolysis and IL-1β production.What’s more, the production of NO inhibits ETC. M2 macrophages can obtain enough ATP from OXPHOS through the TCA cycle. As for energy gain, FAO is essential. HK, hexokinase; Glucose 6-P, glucose 6-phosphate; Fructose 6-P, fructose 6-phosphate; PFK1, 6-phosphofructokinase 1; PK, pyruvate kinase; PPP, pentose phosphate pathway; G6PD, glucose 6-phosphate dehydrogenase; iNOS, inducible nitric oxide synthase; NO, nitric oxide; ETC, electron transport chain; TCA, tricarboxylic acid; OXPHOS, oxidative phosphorylation; IDH, isocitrate dehydrogenase; NADPH, nicotinamide-adenine dinucleotide phosphate; SDH, succinate dehydrogenase; FAS, fatty acid synthesis; FAO, fatty acid oxidation; Acetyl-CoA, acetyl-Coenzyme A; IL-1β, interleukin-1β; HIF-1α, hypoxia inducible factor 1α.

Mills et al. first proposed the notion that arginine can be used to determine the functions of macrophages (119). The M1 macrophages are a product of the iNOS pathway, while the M2 macrophages come from the arginase pathway (60). L-Arginine takes part in initiating of intracellular signaling pathways in macrophages, such as triggering inflammatory responses and accelerating the sensitivity to bacterial endotoxin (120). Arginine deprivation attenuates osteoclastogenesis and also dampens generation of IL-4 induced multinucleated giant cells. Strikingly, in the absence of extracellular arginine, osteoclasts and IL-4-induced multinucleated giant cells display flexibility, since their formation can be restored by supplementation with select arginine precursors *in vitro (* 121). The production of α-Ketoglutarate (αKG) is important for activation of M2 macrophages, including engagement of FAO and epigenetic reprogramming of M2 genes. The potential M2-promoting mechanism is demonstrated by the high αKG/succinate ratio,Whereas a low αKG/succinate ratio strengthens M1 polarization in macrophages (61). Nontargeted metabolomic analysis revealed that tryptophan metabolism is involved in M1 polarization (122). Ornithine decarboxylase (ODC), the rate-limiting enzyme in polyamine synthesis, leads to an increase in putrescine levels and imparis the transcription of M1 genes. In M1 macrophages, the translation of proinflammatory mediators can be regulated by spermidine and spermine (15). In applied research, there are no clear results that using amino acid auxotrophy to decrease the growth of cancerous lymphocyte, though attempting for decades (123). As for macrophages, there is also a long way to go. The detailed metabolic pathways in macrophages are shown in **Figure 3**.

In addition, the classic metabolic pathways, some bypasses, and the enzymes involved have also come into view in recent years. Glucose is converted to sorbitol by aldose reductase (AR) in Polyol pathway. When activating the Polyol pathway, the production of proinflammatory cytokines was increased in kidney cortex of diabetic mice (124). What’s more, upregulation of AR in human macrophages is proinflammatory in foam cells in atherosclerosis (125). When inhibiting the activity of AR, the phenotype was transferred to M2 (126). Ribulose 5-phosphate 3-epimerase (RPE) is an important enzyme for cellular response against oxidative stress and takes part in PPP, contributing to the reversible conversion of D-ribulose 5-phosphate to D-xylulose 5-phosphate (127). The gene coding RPE has exposed to be relative to poor outcome in cancer patients (128). Some metabolites also affect the function and polarization of macrophages. Lactate-derived lactylation of histone serves as an epigenetic modification and could directly stimulates M2 gene expression during M1 macrophage polarization (129). Ketone body, especially, β-hydroxybutyrate (BHB) promotes M2 macrophage polarization, contributing to the resolution of intestinal inflammation and providing ideas for the treatment of inflammatory bowel disease (130). Metabolites in shaping the phenotypes and functions of macrophages were summarized in **Table 2**.

**Table 2:** Metabolites in shaping the phenotypes and functions of macrophages.

Metabolites	Changes in downstream molecules	Changes in phenotypes or functions	Changes in surrounding environment	Ref.
Lactate	lactylation of histone lysine	M1; Arg1 were induced	Promoting wound healing and homeostasis	(129)
	lactylation and acetylation of HMGB1	Changes in exosomes released from macrophages	Increasing endothelium permeability	(131)
	suppressing the NLRP3 inflammasome and the caspase-1 pathway	M2; decreasing the phagocytic capability; suppressing macrophage pyroptosis	Modulating the intestinal microbiota	(132)
Glutamine	Increasing glutamine	M2	Inducing epithelial cell proliferation	(133)
	Inhibiting non-canonical transaminase pathway	Impaired phagocytic capacity	Clearance of dying cells and maintenance of tissue homeostasis	(134)
	High glutamine synthetase	M2	Promoting cancer cells to release N-acetylaspartate	(135)
Ketone body	Addition of BHB	M2	Repairation of mucosa	(130)
		Decreasing the percentage of M1	Suppressing colonic inflammation	(136)

Arg1, arginase 1; HMGB1, high mobility group box-1; BHB, β-hydroxybutyrate.

## Maternal-Fetal Interface Macrophages Remain Enigmatic

Houser (137, 138) found two distinct subsets of CD14^+^ dMϕs in the decidual tissues in early pregnancy: CD11cHI (CD11c high) and CD11cLO (CD11c low). CD11CHI dMϕs express genes related to lipid metabolism and inflammation, and may be the major APCs in the decidua. dMϕs (139) were further analyzed and three subtypes were identifiedin the first trimester of gestation: CCR2^-^CD11cLO (~80%), CCR2^+^CD11cHI (10–15%) and CCR2^-^CD11cHI (~5%). CCR2^-^CD11CHI macrophages were significantly different from the other two subsets in the heat map. CCR2-CD11cHI macrophages are specifically enriched in pathways related to cell metabolism and proliferation. The CCR2^+^CD11cHI subset wasparticularly involvedin the TNF signaling pathway and phagocytosome pathway. Few pathways were particularly enriched in the CCR2^-^CD11cLO subset. In addition, high expression levels incarbon metabolism genes and almost all genes related to heme metabolism, including NADPH,quinone oxidoreductase 1 (NQO1), ferritin heavy chain (FTH1) and HMOX1, were observed in CCR2^-^CD11cHI macrophages. HMOX1 is an enzyme that catalyzes the degradation of heme into carbon monoxide, ferrous iron, and biliverdin. Anti-inflammatory and antioxidant properties have been convincingly demonstrated in the above byproducts of heme metabolism (140). Thus, it is inferred that CCR2^-^CD11cHI macrophages may have an effect of anti-inflammation during the first trimester. Whetherand how the overall metabolic environment affects the function of macrophages are unanswered. Whether there is a special metabolic pattern in decidua macrophages remains to be explored. The association between disorders and metabolism in macrophages is shown in **Table 3**.

**Table 3:** Association between disorders and metabolism and function in macrophages.

Disorders	Main types	Main metabolic changes	Functional phenotypes
Tumors	TAMs (M2) (141, 142)	1) Increased mitochondrial respiration (64); 2) Reduced glycolysis (64)	1) Combination and secretion of galectin-3 (143)
			2) Activation of ROS (143)
			3) Induced HIF-1α (144)
Obesity and Diabetes	(M1) (81, 145)	1) Reducing lipid transportation (146, 147); 2) Increased glycolysis (66)	Production of ROS (148)
Atherosclerosis	M1 (149)	Increased glycolysis (150)	1) Proinflammatory phenotype (149, 150)
			2)Reduced phagocytic activity (151)
			3) Production of foam cells (146, 152)
Injury and Repairation	M1 → M2 (153)	Activation of glycolysis, the TCA cycle, the PPP, FAS and FAO (154)	Transform from proinflammatory to reparative phenotype timely (153, 154)
Pregnancy- related diseases	a mixed group (155, 156)	Still being studied	Dynamic changes of phenotypes, M1 → M1/M2 → M2 (137–139, 156)

TAMs, tumor-associated macrophages; PPP, pentose phosphate pathway; TCA, tricarboxylic acid; FAS, fatty acid synthesis; FAO, fatty acid oxidation.

## Therapeutic Perspective of Intervening in Metabolism *via* Macrophages

The different polarization types of macrophages have an obvious influence on the effects of therapies. At present, there are many ways to regulate the polarization of macrophages, and metabolic regulation is a new research field with great development. Moreover, the research on intervention in macrophages in metabolism as a therapeutic method mainly focuses on tumor-related fields. For example, interference with the phenotypes of TAMs, which are known to be immunosuppressive, is potential to be helpful combing with immunotherapy and/or chemotherapy (157). THP-1 M1 macrophages increased etoposide-induced cancer cell apoptosis, while M2 macrophages decreased apoptosis of cells (158). Recently, researchers found that itaconic acid is one attractive candidate for anti-tumor responses, since peritoneal tumors could be controlled by specifically targeting resident macrophage-associated itaconate levels (159). Although precise regulation is unclear, the targeting of resident macrophages is a potential perspective for future research. Moreover, it was found that administration of miR-223 over-expressed macrophages, with IL-4 preconditioning, attenuated sepsis severity in a LPS model, through the inhibition of the glycolysis pathway. These cells are thus proposed as a candidate for cell therapy during the pro-inflammatory phase of sepsis (160).

Metformin is one of the few drugs that clearly affect macrophage function by regulating its metabolism. Metformin can regulate glycometabolism, and is used as an antidiabetic agent. In metformin treated tumor tissue, M2-like macrophages decrease while M1-like macrophages increase. AMPK-NF-κB signaling, a pathway involved in regulating M1/M2 expression, can be activated by metformin and induce cytokines expression for macrophage polarization to an antitumor phenotype (161). In addition, macrophages could be transformed to foam cells through cholesterol transport and storage. Foam cells are present in all stages of atherosclerosis. Foam cell targeting anti-inflammatory therapies are known to indirectly regulate the actions of pro-atherogenic and anti-atherogenic cytokines (162). What’s more, in a murine model of pancreatic cancer, atorvastatin, a frequently used inhibitor for cholesterol synthesis and HMG-Coa reductase, facilitates TAMs to reprogram to M2-like phenotype and attenuates the chemotherapeutic efficacy of gemcitabine on pancreatic cancer (163).

Regulation of key enzymes in the metabolic process is also one of the main methods of treatment. In a recent trial of non-alcoholic fatty liver disease (NAFLD) (164), acetyl-coenzyme A carboxylase (ACC) inhibitor was used alone or cooperated with a diacylglycerol acyltransferase 2 (DGAT2) inhibitor in patients to observe the change of magnetic resonance imaging - proton density fat fraction in liver. ACC inhibitor monotherapy showed significant anti-liver steatosis, suggesting a potential clinical benefit for chronic treatment. Co-administration of these two trugs is possible to decrease some of the limitations of ACC inhibition alone. It can be seen from this study that the regulation in the metabolic process has the possibility of clinical application. But the influence of key enzymes on the functions of macrophages is still in the stage of scientific research. For example, lactate dehydrogenase (LDH) can catalyse the reversible conversion of pyruvate and lactate in glycolysis. When feeling tumor-derived miR-375, TAMs downregulated the expression of LDHB and increased aerobic glycolysis and lactagenesis (165). Then tumor-derived miR-375 was established as a novel regulator of macrophage metabolism in breast cancer. More research is needed in pregnancy-related diseases. Intervention in metabolism in macrophages is a very rational immunotherapeutic perspective, and it will bring good news for patients.

## Author Contributions

J-XS and X-HX conceived of the manuscript. J-XS wrote the manuscript and prepared the figures. LJ and X-HX reviewed the manuscript. All authors listed have made a substantial, direct, and intellectual contribution to the work and approved it for publication.

## Funding

This study was funded by National Natural Science Foundation of China (81730039, 82071653, 81971384, and 82171657).

## Conflict of Interest

The authors declare that the research was conducted in the absence of any commercial or financial relationships that could be construed as a potential conflict of interest.

## Publisher’s Note

All claims expressed in this article are solely those of the authors and do not necessarily represent those of their affiliated organizations, or those of the publisher, the editors and the reviewers. Any product that may be evaluated in this article, or claim that may be made by its manufacturer, is not guaranteed or endorsed by the publisher.

## Glossary

**Table:**

EMPs	erythro-myeloid progenitors
PB	peripheral blood
iPSC	induced pluripotent stem cell
ICs	immune complexes
ROS	reactive oxygen species
Arg1	arginase 1
MIP	macrophages inflammatory protein
RANTES	regulated on
activation	normal T cell expressed and secreted
MCP-1	monocyte chemo-attractant protein-1
iNOS	inducible nitric oxide synthase
TNF	tumor necrosis factor
TLR	toll-like receptor
TGF‐b	transforming growth factor‐b
LPS	lipopolysaccharide
GM-CSF	granulocyte-macrophage colony stimulating factor
VEGF	vascular endothelial growth factor
ERK	extracellular signal-regulated kinase
EGF	epidermal growth factor
IRF	interferon‐regulatory factors
AMPK	adenosine monophosphate kinase
AP1	activator protein1
NF‐kB	nuclear factor‐kB
PPAR‐g	peroxisome proliferator‐activated receptor‐g
SIRPa	signal regulatory protein a
Tim-3	T cell immunoglobulin and mucin domain-3
PK	pyruvate kinase
HK2	hexokinase 2
PPP	pentose phosphate pathway
PFK1	phosphofructokinase 1
G6PD	Glucose 6-phosphate dehydrogenase
OXPHOS	oxidative phosphorylation
IDH	isocitrate dehydrogenase
NADPH	nicotinamide-adenine dinucleotide phosphate
SDH	succinate dehydrogenase
ACLY	ATP‐citrate lyase
ETC	electron transport chain
IL-1b	interleukin-1b
IRG1	immuneresponsive gene 1
HIF-1a	hypoxia inducible factor 1a
PPR	proton production rate
LDHA	lactate dehydrogenase A
FAO	fatty acid oxidation
FAS	fatty acid synthesis
FASN	fatty acid synthase
PDPK1	phosphoinositol-dependent protein kinase 1
LDs	lipid droplets
NRF2	Nuclear Factor Erythroid 2-Related Factor 2
PUFA	polyunsaturated fatty acid
a-KG	aketoglutarate
TAMs	tumor-associated macrophages
ABCA1	ATP-binding cassette transporter A1
IR	insulin resistance
CPT	carnitine palmitoyltransferase
AT	adipose tissue
FC	free cholesterol
ATMs	adipose tissue-derived macrophages
